# HIV-1 Virus Interactions With Host Proteins: Interaction of the N-terminal Domain of the HIV-1 Capsid Protein With Human Calmodulin

**DOI:** 10.1177/1934578X19849190

**Published:** 2019-05-28

**Authors:** Ywh-Min Tzou, Ronald Shin, N. Rama Krishna

**Affiliations:** 1 Department of Biochemistry and Molecular Genetics, and Comprehensive Cancer Center, University of Alabama at Birmingham, AL, USA; 2 Scott-Ritchey Research Center, College of Veterinary Medicine, Auburn University, Auburn, AL, USA

**Keywords:** HIV-1 virus, calmodulin, capsid protein, N-terminal domain, ITC, NMR

## Abstract

The human immunodeficiency virus (HIV-1 virus) exploits several host factors for assembly, infection, and replication within the infected cells. In this work, we describe the evidence for an interaction of the N-terminal domain of the HIV-1 capsid protein with human calmodulin. The precise role of this interaction within the life cycle of the HIV-1 virus is yet to be defined. Potential roles for this interaction in the viral capsid uncoating are discussed.

The human immunodeficiency virus (HIV-1 virus) exploits a large number of host factors for assembly, infection, and replication within the host cell.^1–8^ Thus, a detailed characterization of these interactions could improve our understanding of the role of these host factors within the HIV-1 life cycle. More importantly, such studies could contribute to the development of antiviral therapies. Several structural and biology studies have already been reported documenting interactions between the HIV-1 virus and host cell proteins; these include the interactions such as cyclophilin/the N-terminal domain (NTD) of the capsid protein,^9^ calmodulin (CaM)/gp160,^10–13^ CaM/matrix protein (MA),^14–18^ CaM/Nef,^19^ lysyl-tRNA-synthetase/the C-terminal domain of the capsid protein,^20^ APOBEC3G/Vif,^21^ DCAF1/Vpr,^22^ importin-α/Vpr,^23^ CD4/ VpU,^24^ Hck/Nef,^25^ and Trim5α/CA,^26^ to name a few.

The current work is focused on a novel interaction we identified between the HIV-1 capsid protein (CA) with calcium-saturated human calmodulin (hCaM) ((Ca^2+^)_4_-CaM). An analysis of the HIV-1 CA sequence using the CaM binding site search algorithm^27^ identified a putative CaM-binding region, IYKRWIILGLNKIV (residues 129–142) with a 1-5-8-14 motif located on helix-7 of the NTD (Figure 1). Figure 2 shows the isothermal titration calorimetric data on the interaction of both the full-length CA protein (as a monomeric mutant) as well as its NTD (CA-NTD) with the (Ca^2+^)_4_-CaM. The stoichiometry of binding for each was 1:1. The full-length monomeric mutant CA binds CaM with a dissociation constant *k*_d_ of 8.13 μM whereas the isolated NTD binds with a *k*_d_ of 3.73 μM. The slightly weaker binding to the full-length protein might be suggestive of small steric hindrance from its C-terminal domain when CaM is binding to the helix-7 on the NTD. To further establish the binding of the CA-NTD to CaM, we examined the ^15^N-HSQC spectra of uniformly ^15^N-labeled CA-NTD without and with excess (1 to 1.75) of (Ca^2+^)_4_-CaM. The data are shown in Figure 3. Some well-resolved native NTD peaks experienced small or zero shifts, but several of the peaks from the native NTD (blue peaks) disappeared and are accompanied by the appearance of several new peaks (green) elsewhere in the spectrum upon the addition of excess (Ca^2+^)_4_-CaM, i.e., they experience rather large shifts. The binding is tight; there was no gradual shift of peaks with increasing additions of CaM. Based on a qualitative examination of the well-resolved peaks in the two spectra (without and with excess CaM), we tentatively conclude the following: the NMR signals from some residues (e.g., E128, Y130, K131, L138, N139, V142, R143) in the 1-5-8-14 motif region in H7 in the free NTD disappear upon CaM binding, and probably show up as new peaks elsewhere in the NMR spectrum. It is also likely that these residues constituting the 1-5-8-14 motif retain a helical conformation in the CaM-bound state as well, as is typical of the sequences with this motif. However, some of the residues (e.g., V126, G127, M144, S146) at the N- and C-terminals of H7 experience some small shifts (instead of disappearing), presumably because they are outside the CaM binding pocket. Some resolved residues from H2 (e.g., F32, S33, E35, I37, M39, S41, A42, L43) disappear (and reappear else-where), suggesting major structural changes in this helix. In H4, some resolved residues (A65, M66, T72, E75, E76, A77, R82, H84) experience small or little shifts, indicating that H4 is probably relatively intact. In H5, the central residue peaks from S102 and D103 show relatively small shifts, but the terminal residues R100 and I104 disappear suggesting partial unwinding. We emphasize that this is just a qualitative interpretation based on shifts (disappearance of peaks, absence of shifts, or small shifts) of a few well-resolved peaks from residues in the NTD and its CaM complex. Because of the significant overlap of the peaks between the free and bound states of NTD, it is difficult to draw unambiguous conclusions from a mere qualitative inspection of the HSQC spectra in Figure 3. Nevertheless, the appearance of several new broad overlapping peaks in the bound state in the general vicinity of ~8.3 ppm (^1^H)/~122 ppm (^15^N) suggests that the binding of CaM to the H7 results in an increase in disordered regions in NTD, likely from the loss of secondary structures of some nearby helices such as H2 (in the helix bundle). Residues in inter-helical loop regions in general showed small shifts.

When titrated with apo-CaM from 0% to 20%, no significant shifts were observed in the NTD heteronuclear single quantum coherence (HSQC) spectra.

To further confirm that the (Ca^2+^)_4_-CaM is indeed binding to the H7 containing the sequence with the 1-5-8-14 CaM-binding motif, a peptide (unblocked) with the sequence PVGEIYKRWI ILGLNKIVRMYS from the NTD was synthesized. The HSQC results of the peptide binding to ^15^N-CaM are shown in Figure 4 (0.25 mM CaM; 0.4 mM peptide), confirming that CaM is probably recognizing and binding to the CaM-binding motif in the H7 helix of the CA-NTD.

In Figure 5, we show the ^15^N-HSQC spectra of uniformly ^15^N-labeled (Ca^2+^)_4_-CaM, without and with NTD, again confirming the interaction between the two proteins. Noteworthy is that the shifts observed in Figure 4 (with the peptide) and Figure 5 (with NTD) are somewhat similar, suggesting similar types of intermolecular contacts in both the CaM/peptide and CaM/NTD complexes.

Our ITC and NMR results in this work demonstrating an interaction between the CA-NTD of HIV-1 and (Ca^2+^)_4_-CaM are in disagreement with the earlier work of Radding et al.^14^ who used ^125^I-labeled CaM overlays of sodium dodecyl sulfate-polyacrylamide gels and detected interaction of CaM with the Gag and p17 (matrix protein) but not with the p24 (the capsid protein). It may be likely that their negative result is an artifact of the particular assay used by them. NMR spectroscopy is an exquisitely sensitive technique in detecting protein-protein and protein-small molecule interactions over a rather wide range of binding conditions (very weak to very tight).

Following the identification of the CaM-binding motif in the HIV-1 capsid sequence, we examined the capsid sequences of a few other retroviruses for the presence of CaM-binding motifs. Interestingly, the results do not show a uniformity in the location of the CaM-binding motifs among the sequences we examined. For example, the CaM-binding motif is located on helix-7 of the NTDs of HIV-1 and EIAV, on helix-1 of the NTDs of RSV and BLV, on helix-4 of MLV, and most interestingly, it is totally absent from the capsid proteins of HTLV-1 and HTLV-2. This non-uniformity of the location of the CaM-binding motifs in the capsid proteins of these viruses suggests that CaM may not share a mode of action and functional role that is common to the capsid proteins of these retroviruses. It may be that the role of CaM in interacting with the retroviral capsid proteins may be specific for each virus.

CaM, a calcium-binding protein, is ubiquitously distributed in eukaryotic cells. It binds to a rather large number of target proteins and regulates their activities in response to Ca^2+^ signals.^30,31^ The association of CaM with the HIV-1 life cycle has been established long time ago even though the precise mechanisms are still being defined. The level of CaM increases in cells expressing the HIV-1 envelope glycoprotein.^14^ It has been shown that CaM binds to the gp160,^10–13^ the MA,^14–18^ and the Nef^19^ proteins of HIV-1. The precise role of the interaction of CaM with the NTD of CA within the life cycle of the HIV-1 virus in an infected cell remains unknown. Recombinant HIV-1 CA proteins are known to spontaneously associate *in vitro* to form capsid-like structures.^32^ One consequence of CaM binding to the NTDs of the CA proteins is that such an interaction could potentially inhibit or interfere with the NTD interactions necessary for the formation of capsids or capsid-like structures. Thus, firstly, it is intriguing to think that the HIV-1 virus might potentially exploit the host cell CaM to play a role in the viral capsid uncoating process in the infected cell, e.g., CaM could bind to the NTDs of the newly released CA proteins from the capsid during the uncoating process, discourage these CA molecules from re-associating with the partially uncoated capsid or from self-associating, and thereby facilitating a unidirectional uncoating process. Recent cryoelectron tomography studies revealed the flexible nature of the conical HIV-1 capsid which might potentially accommodate its interactions with host cell factors.^33^ Thus, secondly, it will be interesting to see if CaM can also directly access and bind to the H7 helix of the CA-NTDs in the NTD hexameric and pentameric rings in intact mature capsids. Such a binding could potentially play a role in initiating the viral capsid uncoating process in the infected cell. CaM could also potentially interact with the CA-NTD in the Gag polyproteins, though the role of such an interaction is not clear. The oligomerization of the Gag polyproteins at the plasma membrane of the infected cell leads to the eventual budding of the immature virions. Additional investigations are needed to define the role of CaM/CA-NTD interactions within the life cycle of the HIV-1 virus.

## Experimental

### Preparation of Proteins

The uniformly ^15^N and ^15^N/^13^C labeled and unlabeled proteins were prepared as described in our previous studies.^34,35^

### Isothermal Titration Calorimetry

Full length double mutant (W184A/M185A) HIV-1 capsid protein (WM-CA) was treated with 1 mM DTT in 25 mM NaCl and 25 mM Na-Acetate (pH 5.5) at 37°C for 48 hours. This disulfide-bond-free capsid protein, its NTD and hCaM were then dialyzed with two changes of 25 mM sodium acetate pH 4.7 buffer with 0.2 mM Tris(2-carboxyethyl) phosphine (TECP), 5 mM CaCl_2_, and 25 mM NaCl. After protein content assay with Bradford, the concentrations of proteins were adjusted to 500 nM for hCaM, and 50 nm for full length and N-terminus of HIV1-CA. Proteins were then loaded onto VP-ITC (MicroCal) with incubation temperature of 37°C for binding analysis. CaM (500 nM) was in the injector, and added to the Cell in increments. The cell contained the HIV protein (NTD or WM-CA) at 50 μM. 20 03bcL of CaM solution was injected at each data point.

### NMR Measurements

The 2D/3D-NMR data on the uniformly labeled proteins were collected at 309 K on a Bruker-Biospin Avance III HD 850 MHz NMR spectrometer with a TCI Cryoprobe. The spectra on some initial samples were collected on an Avance III HD 600 MHz NMR system with a TCI CryoProbe. The NMR samples typically contained 10 mM bis-Tris (pH 6.3), 30 μM CaCl_2_, and 10% D_2_O, and protein concentrations ranging from 0.25 mM to 1 mM.

## Figures and Tables

**Figure 1.**
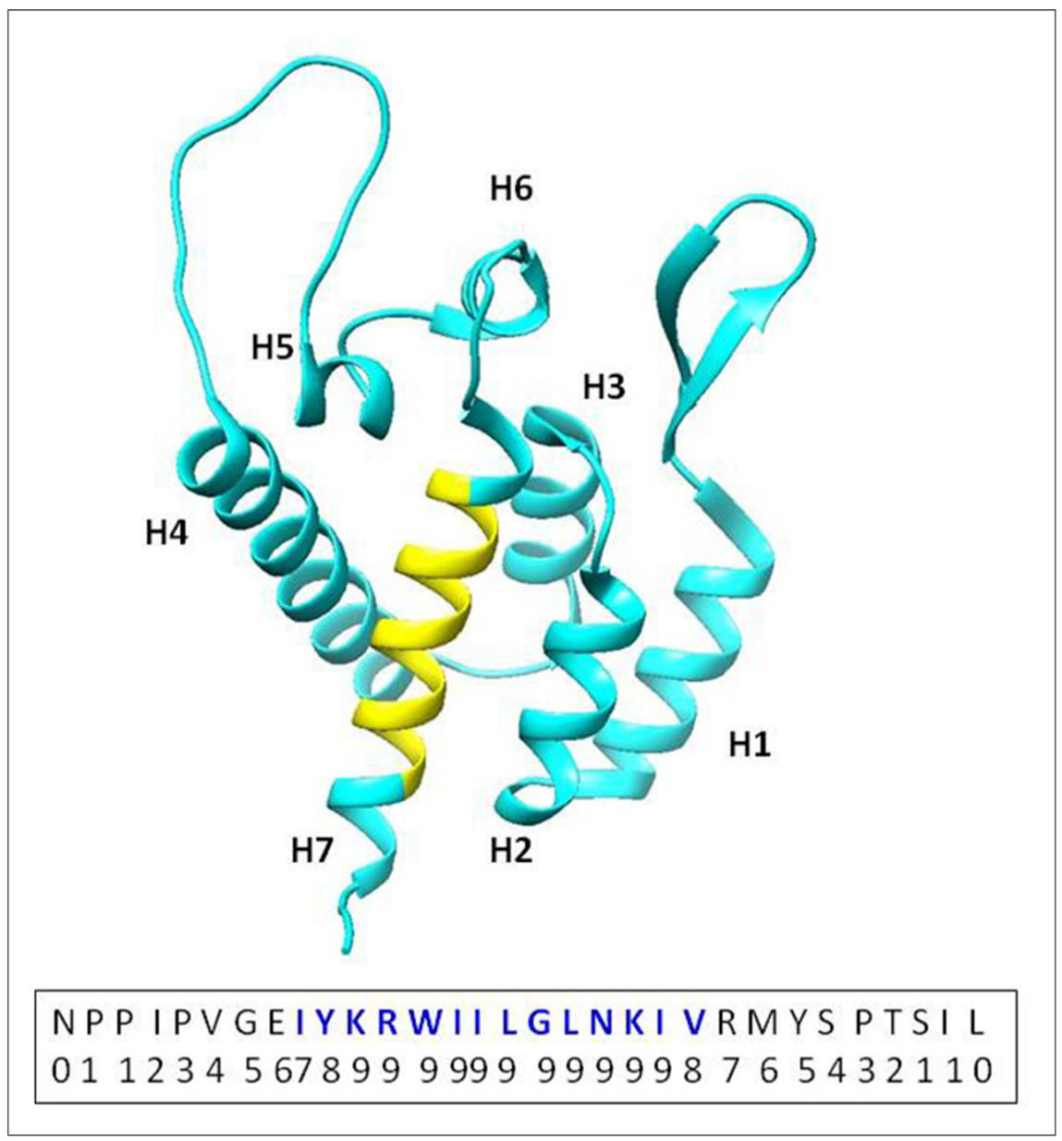
The N-terminal domain (PDB ID: 1GWP^28^) of the HIV-1 capsid protein. The 7 helices, H1 to H7, are identified. The yellow sequence on H7 identifies the calmodulin (CaM)-binding region IYKRWIILGLNKIV with the 1-5-8-14 motif (blue letters). The inset at the bottom shows the sequence of residues 121 to 151 with normalized scores at the bottom for CaM binding with the 1-5-8-14 motif (blue letters). Model created by UCSF Chimera.^29^

**Figure 2.**
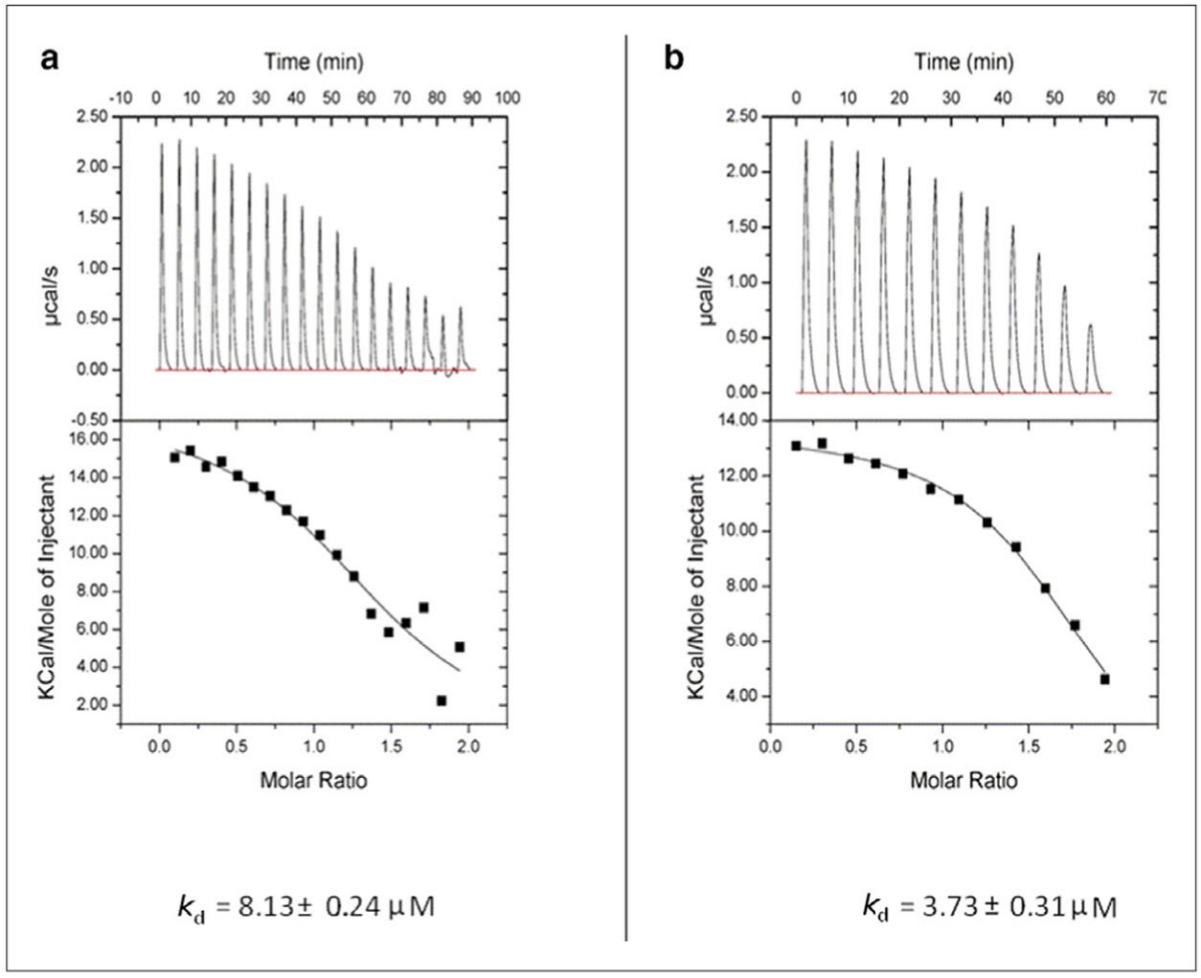
Isothermal titration calorimetry data showing interaction between human calmodulin (hCaM) and HIV-1 capsid protein (CA). (a) hCaM binding to full-length monomeric mutant (W184A/M185A)-CA. (b) hCaM binding to the N-terminal domain of CA. The dissociation constants (*k*_d_) are indicated at the bottom. The stoichiometry of binding in each panel was 1:1.

**Figure 3.**
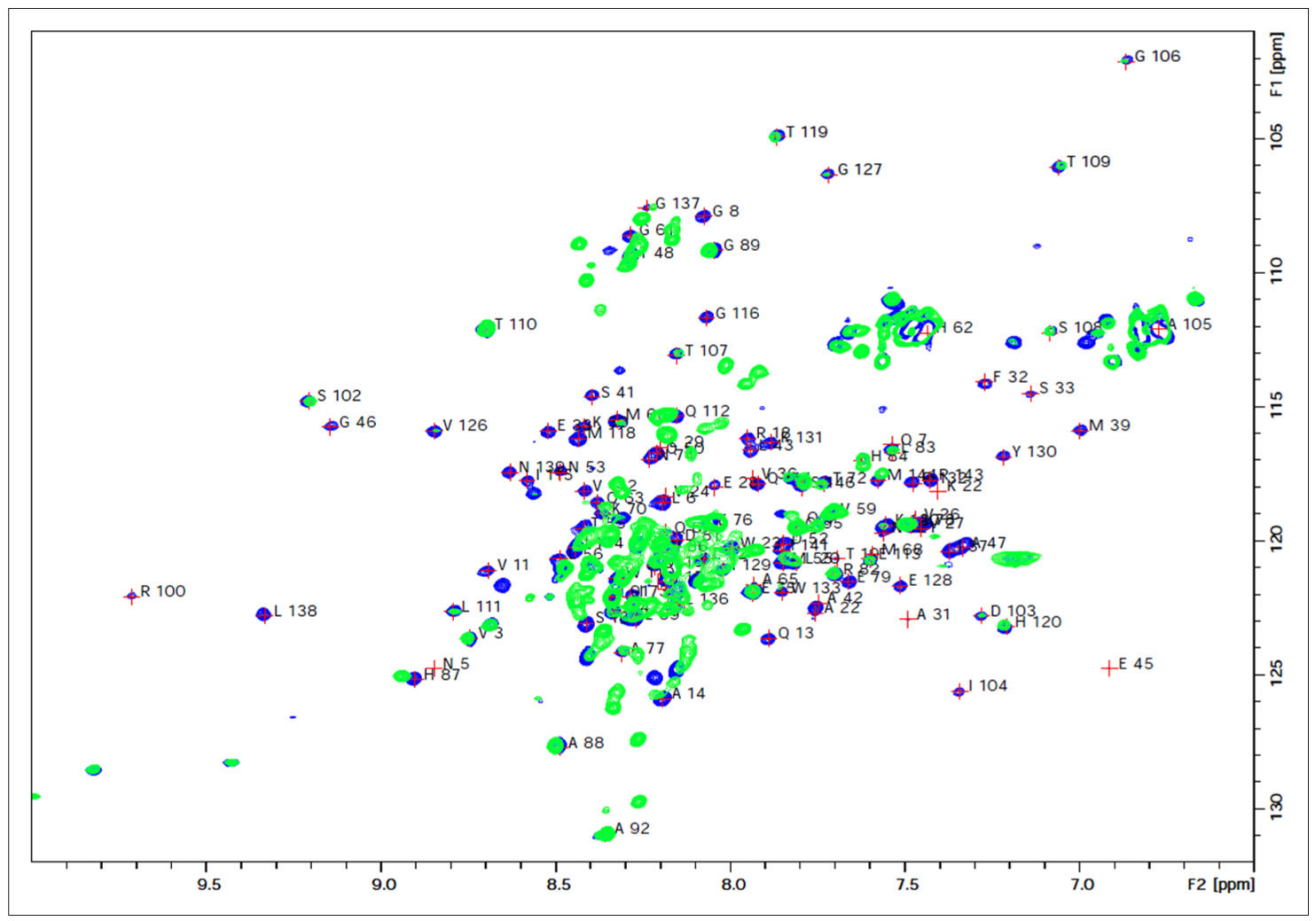
^15^N-HSQC spectrum of the N-terminal domain (NTD) of HIV-1 capsid protein, without (blue) and with excess (1 to 1.75) calcium-bound calmodulin (green). The assignments for the free NTD are also shown.

**Figure 4.**
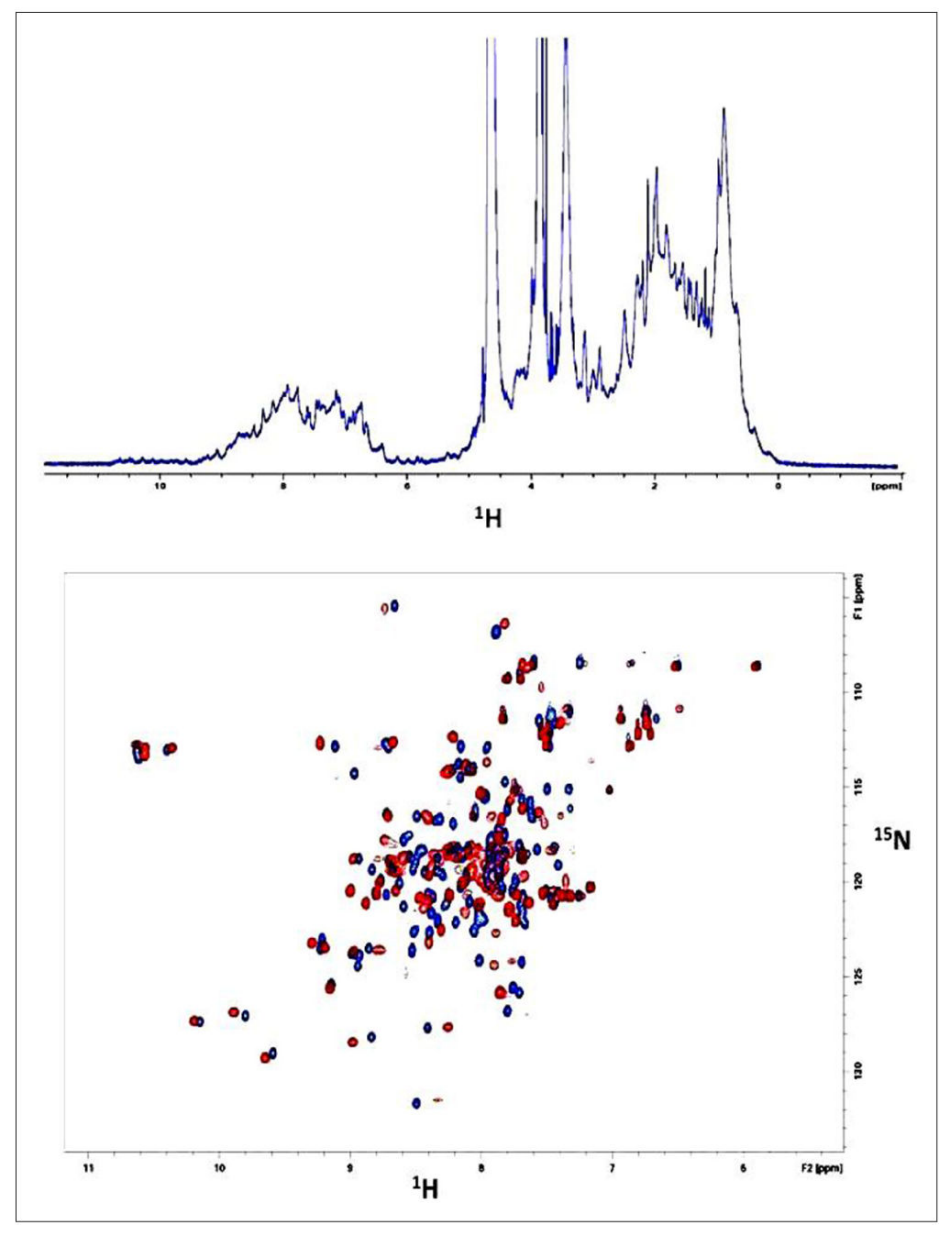
(Top) The 1D-NMR spectrum (without ^15^N-decoupling) of ^15^N-hCaM with the peptide. (Bottom) The corresponding ^15^N-HSQC spectra of uniformly labeled calcium-bound hCaM without (blue) and with (red) excess (0.25 mM CaM; 0.4 mM peptide) of the synthetic peptide representing the CaM-binding sequence PVGEIYKRWIILGLNKIVRMYS in the H7 helix of the NTD of the HIV-1 capsid protein.

**Figure 5.**
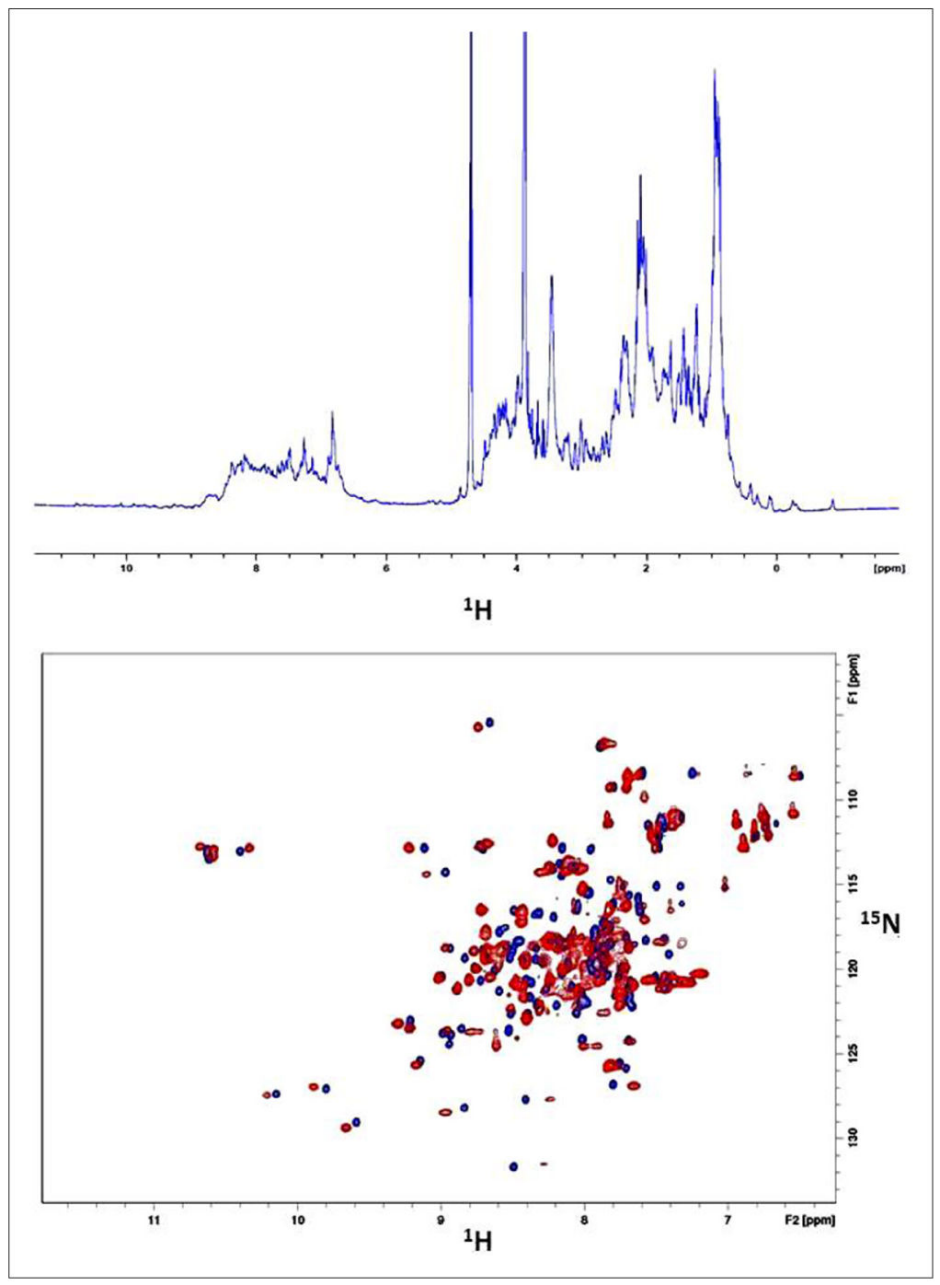
(Top) The 1D-NMR spectrum (without ^15^N-decoupling) of ^15^N-hCaM with the NTD. Some ring-current shifted peaks of the NTD are visible at the high-field end. (Bottom) The corresponding ^15^N-HSQC spectra of uniformly labeled calcium-bound hCaM without (blue) and with (red) a slight excess (1 to 1.1) of the NTD of HIV-1 capsid protein.
